# Designing an Experimental Platform to Assess Ergonomic Factors and Distraction Index in Law Enforcement Vehicles during Mission-Based Routes

**DOI:** 10.3390/machines12080502

**Published:** 2024

**Authors:** Marvin H. Cheng, Jinhua Guan, Hemal K. Dave, Robert S. White, Richard L. Whisler, Joyce V. Zwiener, Hugo E. Camargo, Richard S. Current

**Affiliations:** National Institute for Occupational Safety and Health, Morgantown, WV 26505, USA

**Keywords:** driver–vehicle interface, driving simulator, machine learning

## Abstract

Mission-based routes for various occupations play a crucial role in occupational driver safety, with accident causes varying according to specific mission requirements. This study focuses on the development of a system to address driver distraction among law enforcement officers by optimizing the Driver–Vehicle Interface (DVI). Poorly designed DVIs in law enforcement vehicles, often fitted with aftermarket police equipment, can lead to perceptual-motor problems such as obstructed vision, difficulty reaching controls, and operational errors, resulting in driver distraction. To mitigate these issues, we developed a driving simulation platform specifically for law enforcement vehicles. The development process involved the selection and placement of sensors to monitor driver behavior and interaction with equipment. Key criteria for sensor selection included accuracy, reliability, and the ability to integrate seamlessly with existing vehicle systems. Sensor positions were strategically located based on previous ergonomic studies and digital human modeling to ensure comprehensive monitoring without obstructing the driver’s field of view or access to controls. Our system incorporates sensors positioned on the dashboard, steering wheel, and critical control interfaces, providing real-time data on driver interactions with the vehicle equipment. A supervised machine learning-based prediction model was devised to evaluate the driver’s level of distraction. The configured placement and integration of sensors should be further studied to ensure the updated DVI reduces driver distraction and supports safer mission-based driving operations.

## 1. Introduction

Driving distraction is a critical safety issue for both the general public and law enforcement officers (LEOs). As technology continues to advance, vehicles are increasingly equipped with various instruments and devices designed to aid in performing duties. However, these devices also pose substantial distraction risks, particularly for LEOs who must frequently interact with these devices while driving. Analyzing naturalistic driving data, driver-related factors—such as error, impairment, fatigue, and distraction—accounted for nearly 90% of crashes. Specifically, distraction was a factor in 68% of the 905 analyzed crashes resulting in injuries and property damage [1]. Given the unique responsibilities and the high-pressure nature of their work, it is crucial to understand and mitigate factors contributing to driving distraction in law enforcement contexts.

In the United States, approximately 800,000 LEOs serve in various capacities, including sheriffs and deputy sheriffs, state troopers and highway patrol, and transit and railroad police. Over the past decade (2014–2023), a total of 1387 federal, state, and local LEOs died in the line of duty. Of these fatalities, 547 LEOs were killed in firearms-related incidents, while 496 were killed in traffic-related incidents [2]. In 2022, the rate of fatal work injuries for LEO was 33.25 per 100,000 full-time equivalent workers, compared to 5.3 for all occupations [3]. Additionally, the nonfatal injury rate for LEOs was three times higher than the rate for other U.S. workers, with transportation incidents being the third leading cause of nonfatal injury events in the law enforcement community [4].

Traffic-related incidents have consistently been a leading cause of fatal injuries for LEOs, accounting for 28% of all officer fatalities [2] in 2023. Between 2019 and 2023, a total of 131 LEOs died in fatal automobile crashes, excluding other traffic-related fatalities such as motorcycle crashes and struck-by incidents. Among LEO nonfatal injuries, the median days away from work in 2018 were 10 days, compared to 9 days for all occupational groups in the same year [5]. The economic impact of motor vehicle-related deaths and injuries is significant. A study by the Network of Employers for Traffic Safety showed that the employer’s costs were USD 751,382 per fatality and USD 75,176 per nonfatal injury. Among different reasons that cause traffic accidents, distracted driving has been recognized as a major cause of injuries and deaths among the general public. The total employer cost of distracted driving crashes was USD 18.8 billion in 2018 [6].

Driver distraction [7] is a common occurrence for LEOs in their daily work. This distraction is defined as “diversion of attention from activities critical to safe driving for the performance of a secondary competing activity.” LEOs face a significant risk of distraction due to the nature of their jobs, including high-speed chases, performing maneuvers at high speeds, and the constant need to use and monitor technology in their vehicles [8]. Prior studies have investigated the distraction caused by the human–machine interface in different vehicles [9–11]. Driver distraction from interactions with advanced technologies has been identified as the leading cause of LEO vehicle crashes, contributing to up to 25% of these incidents. During their mission routes, LEOs may need to perform various tasks other than driving, such as checking license plates on mobile data terminals (MDTs), communicating with dispatch via radio, and controlling their vehicle’s sirens. These tasks introduce visual, auditory, and cognitive distractions that can compromise their safety [12]. Prolonged performance of secondary tasks while driving can lead to information overload for officers [13].

Research on driver distractions in both commercial and residential vehicles [10,14] has highlighted several factors that can cause distraction, including complex interfaces [15], usage of mobile devices [16], and frequently updated information from in-vehicle information systems [17]. Although warning messages were designed in the in-vehicle interface for safety considerations, poor design of DVIs can introduce additional visual workload to drivers. Therefore, DVIs need to incorporate drivers’ ability to recognize and react safely to the received warning messages to minimize cognitive distractions. Unfortunately, LEOs must handle an extensive amount of information from the in-vehicle devices on their mission-based route, making the layout of DVIs important to reducing potential distraction.

Studies on MDT use among police have shown that 77% of officers use MDTs while driving, 55% use them while performing one other task, 11% use them while performing two other tasks, and 7% use them while performing three other tasks [18]. The dangers of driver distraction for LEOs are well documented, and some police departments have implemented policies to address these risks. These policies may restrict or even prohibit the use of MDTs while driving at high speeds.

Prior studies have focused on the design and analysis of in-vehicle technologies to improve officer safety [19,20]. An important step in this process was the extensive survey conducted by the Anthropometry Lab at NIOSH, which collected twenty body dimensions (with and without gear) of 756 male and 218 female LEOs through a stratified national survey [21]. This comprehensive data collection facilitated the development of digital LEO body models for various design applications to enhance LEO equipment. The resulting digital models are crucial for ensuring that DVIs are ergonomically optimized. These digital models can then be used in simulated environments to design or improve equipment for LEOs. For instance, the National Institute for Occupational Safety and Health (NIOSH) utilized an anthropometric approach to design tractor rollover protective structures using digital manikins. This approach included data from 100 farm tractor operators, 3718 respirator users, 951 firefighters, and 816 civilian workers [22]. In addition to these efforts, a potential avenue for enhancing officer safety is the use of advanced DVIs that can alleviate some of the vehicle control responsibilities for LEOs.

The development of a driving simulator for law enforcement vehicles represents a significant advancement in addressing these challenges [23]. In particular, an LEO must interact with multiple devices once a mission is assigned, with some interactions being necessary while driving. A well-designed simulator can replicate the complex driving scenarios and diverse tasks that officers encounter in the field, providing a controlled environment for assessing and improving their driving performance. Such a simulator allows for comprehensive testing and training without the associated risks of real-world driving, enabling law enforcement agencies to better prepare their officers for the demands of their roles.

Furthermore, such a platform can be utilized to simulate various scenarios, helping law enforcement agencies to simplify their interaction process and avoid potential accidents caused by managing multiple devices simultaneously. Cutting-edge technologies like automated driving [24] and AI-assisted operations [25,26] can also undergo testing within the driving simulator to assess efficacy in assisting LEOs with their duties and ensuring safer driving conditions. This study aims to develop a unified driving simulator platform, and the primary goal is to capture and analyze various aspects of driver behavior, including reactions to specific stimuli, external distractions, and the execution of control commands under pressure. A variety of data acquisition techniques that combine the driver responses to different stimuli are synthesized for specific driving conditions [27]. By evaluating the acquired results of these factors, the simulator can identify how distractions occur and assess the potential effectiveness of different DVI designs for empirical study.

It also serves as a valuable tool for research and development, facilitating the iterative testing of novel technologies and configurations within a virtual environment prior to their implementation in actual law enforcement vehicles. This proactive approach paves the way for the design of safer and more efficient DVIs tailored to the unique demands of law enforcement operations. By enabling rigorous testing in a simulated space, potential issues can be identified and addressed early on, mitigating risks and enhancing operational readiness. Furthermore, the simulator allows for the exploration of innovative human–machine interface concepts, ensuring intuitive and seamless interactions between officers and in-vehicle technologies. Ultimately, this robust simulator may support optimal law enforcement vehicle systems, enhancing officer situational awareness and bolstering the overall safety and effectiveness of critical field operations [28,29]. This activity was reviewed by the CDC, deemed research not involving human subjects, and was conducted consistent with applicable federal law and CDC policy (§See, e.g., 45 C.F.R. part 46; 21 C.F.R. part 56; 42 U.S.C. §241(d), 5 U.S.C. §552a, 44 U.S.C. §3501 et seq).

In summary, establishing an adequate driving simulator for law enforcement vehicles is crucial for understanding and mitigating the risks associated with driver distraction caused by poor DVIs. This research on law enforcement driving simulators enhances our comprehension of the distractions caused by improper DVIs and provides practical tools and recommendations to improve the design of DVIs. These improvements aim to minimize the risks to LEOs, thereby enhancing their safety on the road.

## 2. System Configuration of Experimental Platform

**Figure 1.**
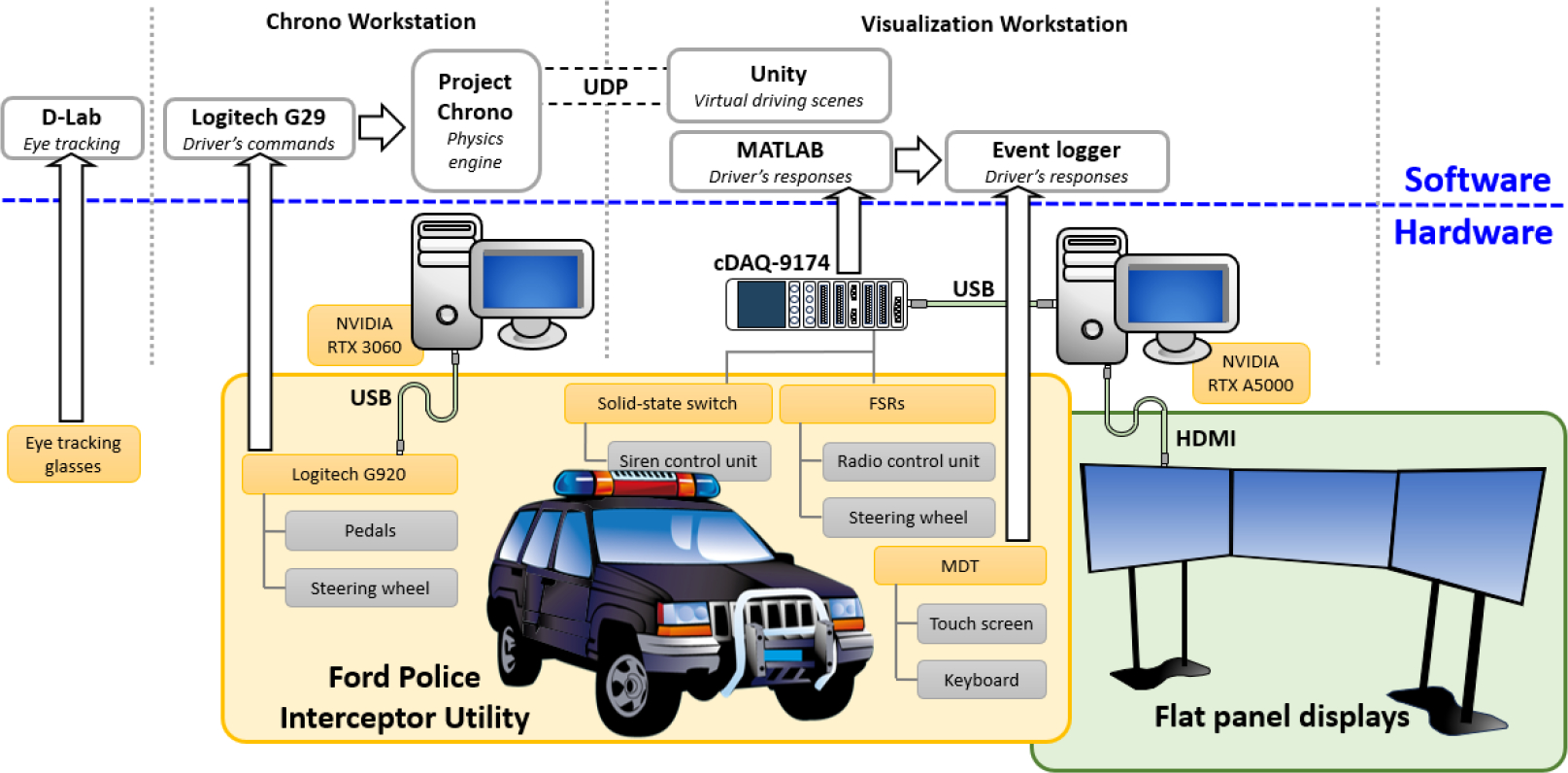
Schematic diagram of the experimental driving simulator platform.

The primary goal of this study was to develop an integrated driving simulator platform that emulates various law enforcement scenarios. To achieve this, an experimental platform capable of capturing driver’s behaviors, including responses to specific stimuli and external distractions, vehicle control commands under pressure, and recording communication during mission-based routes was needed. This development necessitated the careful selection and design of hardware and software components in individual subsystems, such as the data acquisition system, physics emulation system, animation rendering system, and the physical vehicle platform. Figure 1 illustrates the integration diagram of these individual subsystems, demonstrating how each component interacts to create a cohesive and functional simulator environment. The system in this study was designed to ensure that the various elements worked together to emulate accurate and realistic law enforcement driving scenarios.

In this experimental platform, two computers were utilized: an HP workstation with an i9–10900X processor, referred to as the Chrono Workstation, and a Dell workstation with an i9–13900 processor, referred to as the Visualization Workstation. The Chrono Workstation is responsible for receiving the driver’s command inputs and the calculations performed by the physics engine, Project Chrono. The Visualization Workstation manages the signals acquired from the equipped sensors, logs events, and handles animation and visualization implementation, with Unity and the corresponding driving scene installed on it. The physical responses of the selected law enforcement vehicle were calculated on the Chrono Workstation based on the acquired driver input and vehicle dynamics. The calculated results, such as the vehicle’s location, speed, and pose, as well as the road conditions and climate status, were then transmitted to the Visualization Workstation via a User Datagram Protocol (UDP) connection. An event logger implemented on the Visualization Workstation recorded responses not used for vehicle control to capture incidents for driver behavior and distraction analysis. These events are recorded by an event logger and can be used for driver’s behavior and distraction analysis. This setup ensures that data processing and graphical rendering are managed efficiently, allowing for a smooth and immersive driving simulation experience. The following sections describe the selected hardware components, the developed software, and the integration of individual subsystems to create a comprehensive driving simulator system for the law enforcement vehicle.

## 3. Hardware Components

### 3.1. Vehicle Used for the Platform

The experimental setup utilized a retired 2013 Ford Police Interceptor Utility vehicle, originally designed for law enforcement purposes. This vehicle’s reinforced body structure is capable of withstanding high-speed pursuits and minor collisions, typical in police operations. It is also equipped with heavy-duty steel wheels, high-performance tires, and upgraded brakes, factory-installed spotlights, bull bars, and roof-mounted light bars, essential for law enforcement duties. Its high ground clearance and robust suspension system make it suitable for a wide range of terrains, from urban streets to rural backroads.

**Figure 2.**
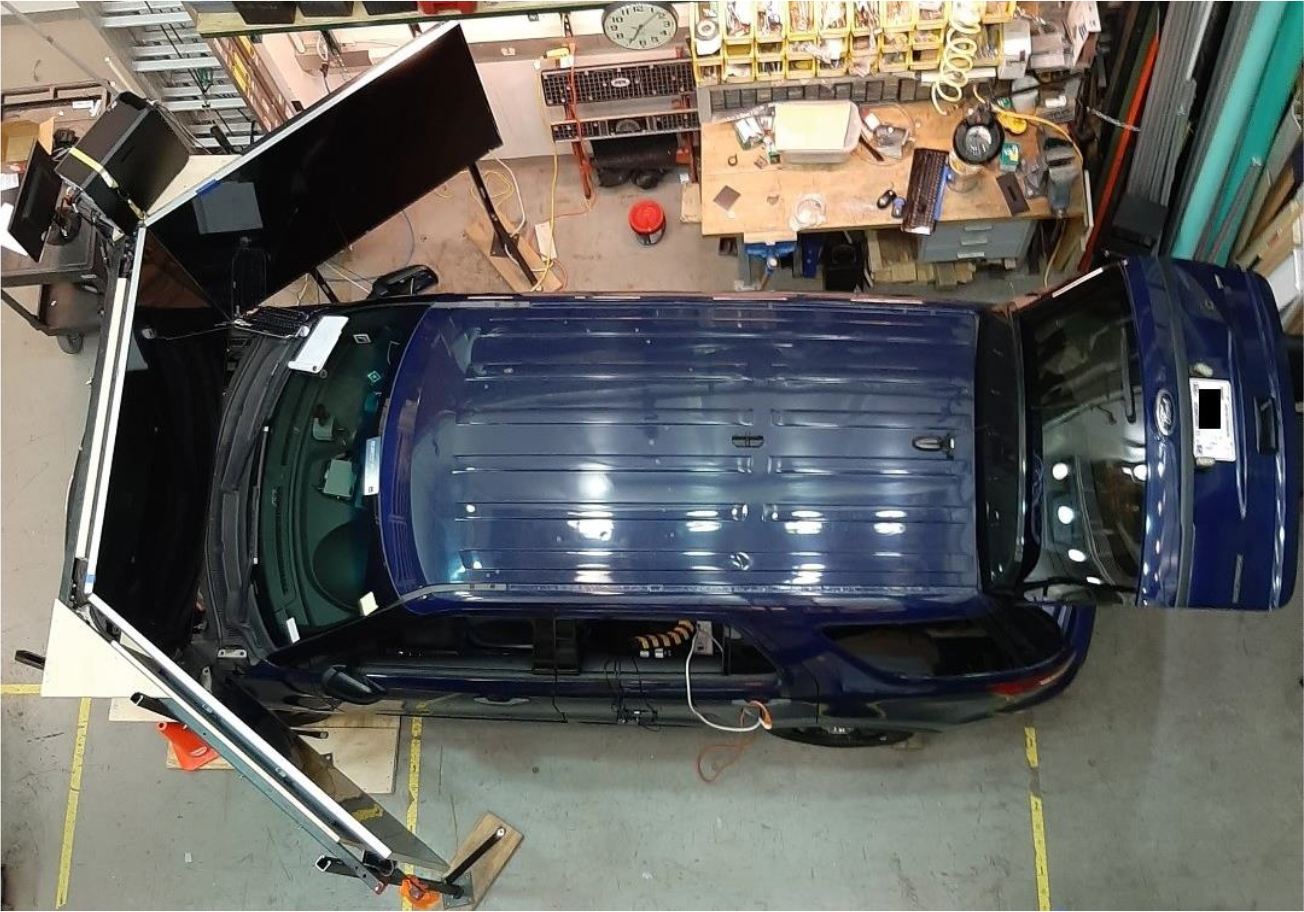
System setup of the virtual environment using a Ford Police Interceptor Utility in a lab setting.

This vehicle is powered by a 3.7-L V6 engine, which is paired with a six-speed automatic transmission. The rear cargo space provides ample room for installing the data acquisition system for this study. Dashboard mounts are reinforced to accommodate police-specific electronics like radios and computers. Interior lighting is optimized for night operations, with red and white LED lights minimizing glare to preserve officers’ night vision. These features were leveraged to monitor and collect data on human behaviors during law enforcement operations. Because this retired law enforcement vehicle adhered to rigorous design and usability standards, it was an ideal platform for our research. Figure 2 illustrates the experimental setup of the driving simulator platform.

### 3.2. Instruments Installed in the Experimental Law Enforcement Vehicle

Law enforcement vehicles are usually equipped with a variety of specialized instruments and equipment designed to assist officers in performing their duties effectively and safely. Most of the equipment was removed to support the following instruments installed on the experimental platform. The instruments installed in the current platform include the following:

- Two-Way Radio: Essential for communication with dispatchers and other officers;
- Mobile Data Terminal (MDT): A rugged laptop used for accessing databases, running license plate checks, and receiving dispatch information. The original laptop was replaced by a regular touchscreen laptop in the experiments;
- Dash Camera: Records video of the vehicle’s front view;
- Sirens: The sirens installed in the engine compartment serve to augment the vehicle’s auditory signaling capabilities to alert surrounding traffic and pedestrians;
- Rear Seat Camera: Monitors the back seat area where detainees are held;
- Radar detector: Detects the speed of oncoming or receding vehicles using radar waves. It is typically mounted on the dashboard or integrated into the vehicle’s front grille, providing real-time data to assist in traffic enforcement and speed monitoring;
- Storage Compartments: Designed for storing additional gear and tactical equipment.

**Figure 3.**
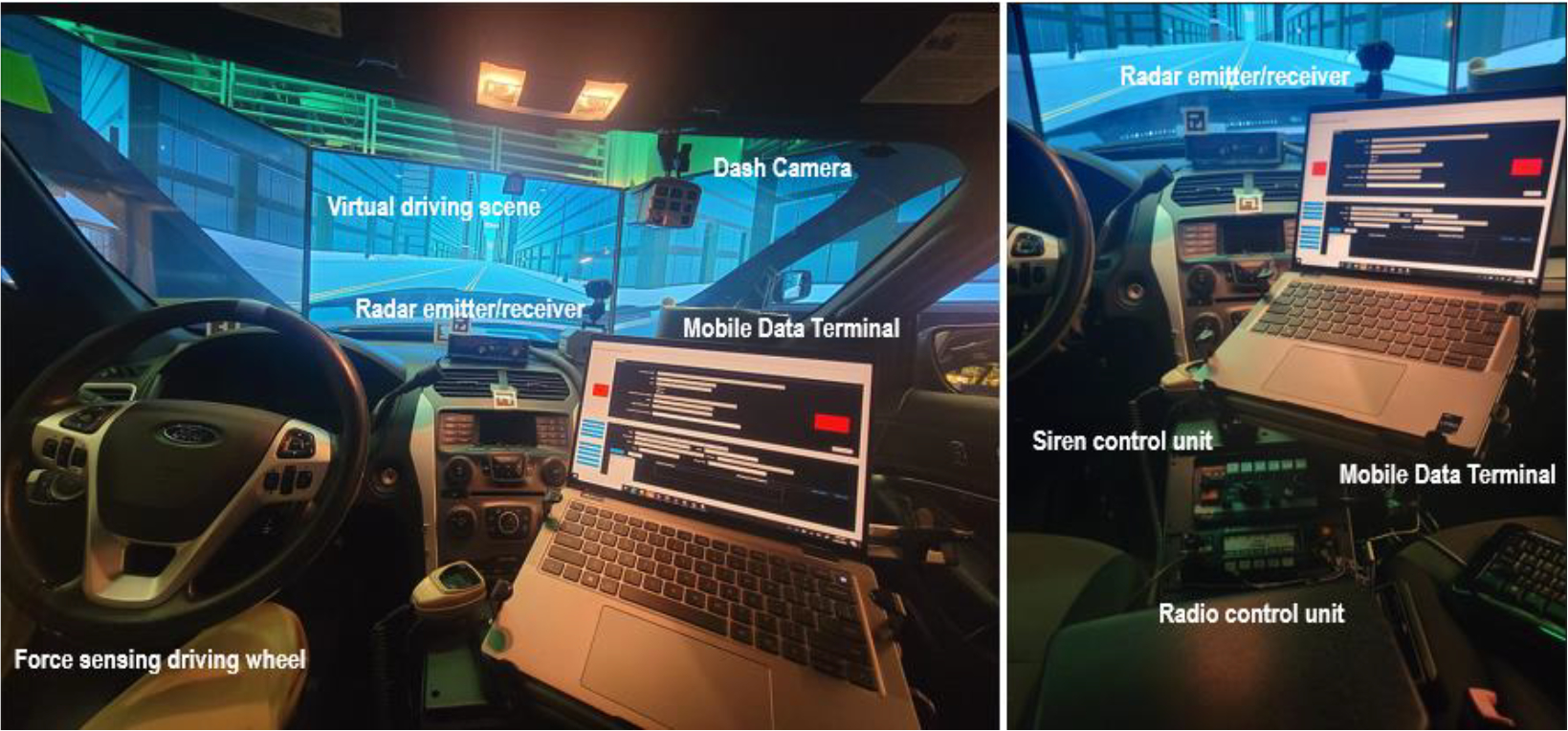
Internal setup of the emulated law enforcement vehicle experimental platform.

These instruments and equipment collectively emulate a law enforcement vehicle. Figure 3 illustrates the internal setup of the experimental platform. Some standard law enforcement equipment, such as lightbars, spotlights, public address (PA) system, body-worn camera interface, vehicle tracking system, automatic license plate reader (ALPR), and weapon mounts, were removed to convert the vehicle for non-law enforcement purposes. In addition to the typical instruments found in a law enforcement vehicle, we installed an eye-tracking system and force-sensing devices to monitor the driver’s attention and measure the force applied to the wheel and paddles. The original driving wheel and paddles were modified with electronic sensors, allowing their movements to be captured by the data acquisition subsystem.

The locations of the equipped instruments, or the Driver–Vehicle Interface (DVI), were carefully considered to minimize interference with or distracting driver’s attention while LEOs are on their mission-based routes.

### 3.3. Configuration of Driver–Vehicle Interface

The DVI can contribute to driver distraction while a LEO is driving on task-based routes [13]. Specifically, officers sometimes need to interact with multiple instruments for designated missions within the limited space of the cockpit. Although not preferred or intended, this interaction may occur while the vehicle is moving. Poorly designed DVIs, especially when fitted with aftermarket police equipment, can lead to perceptual-motor problems such as obstructed vision, difficulty reaching controls, and operational errors. These issues can significantly distract the driver. Therefore, the locations of the instruments, as mentioned in the previous section, need to be carefully arranged.

**Figure 4.**
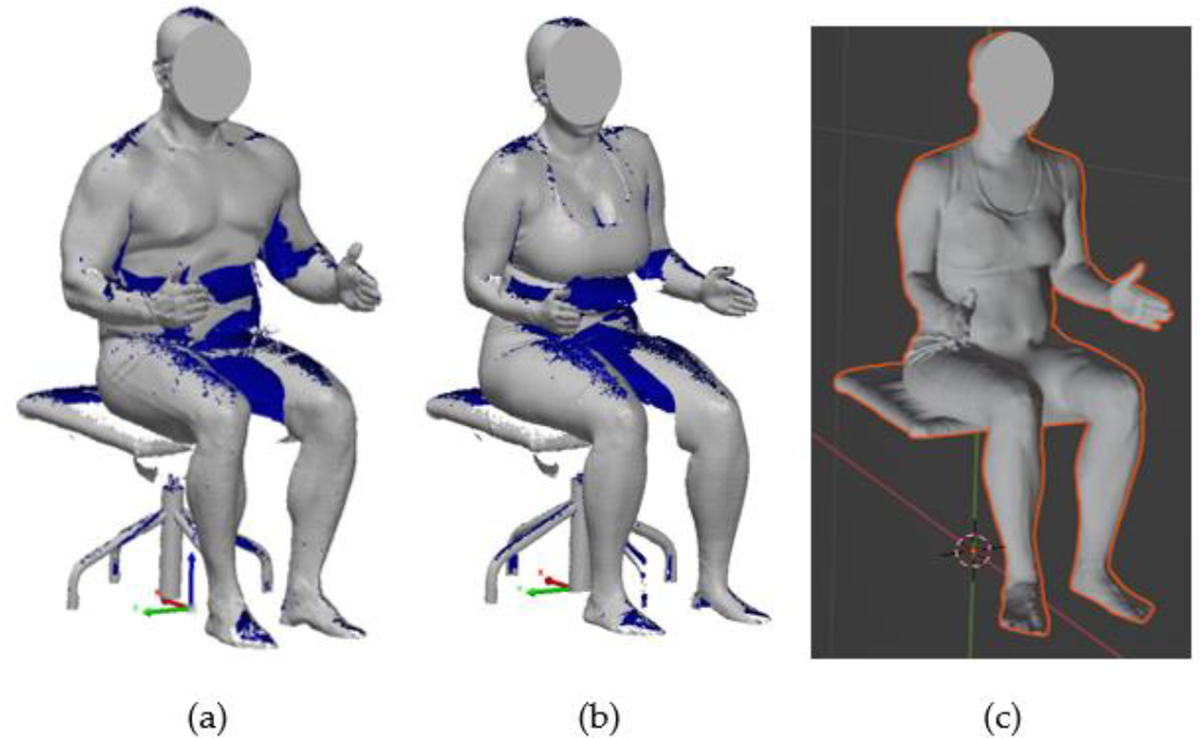
Comparison of digital models between scanned model and the rendered model of LEOs: (**a**) a raw scanned model of an average male LEO, (**b**) a raw scanned model of an average female LEO, (**c**) a rendered model of a female LEO used for emulation in Blender. The raw scanned models contain missing data (blue) that must be post-processed before rendering.

NIOSH has gathered extensive body dimension data for LEOs across the United States through a comprehensive national survey [30,31]. This survey involved collecting twenty body dimensions, both with and without gear, from 756 male and 218 female LEOs using a data collection trailer that traveled nationwide. Over the past few years, the Human Factors Team at NIOSH has applied a Multivariate Anthropometric Models Analysis approach to develop digital body models of LEOs [32]. Typically, LEOs tend to be heavier and have a larger upper torso build compared to the general civilians. Figure 4 demonstrates the scanned models of LEOs derived from previous studies. Previous research identified fifteen men and fifteen women representing the unique body size and shape composition of the LEO population. This led to the creation of a combined set of 24 digital body models in three-dimensional form, removing six redundant models where female and male body shapes overlapped. These digital models, along with their detailed anthropometric measurements, are instrumental in facilitating the design of LEO cruiser cabs, ensuring they accommodate the diverse body dimensions of officers effectively.

**Figure 5.**
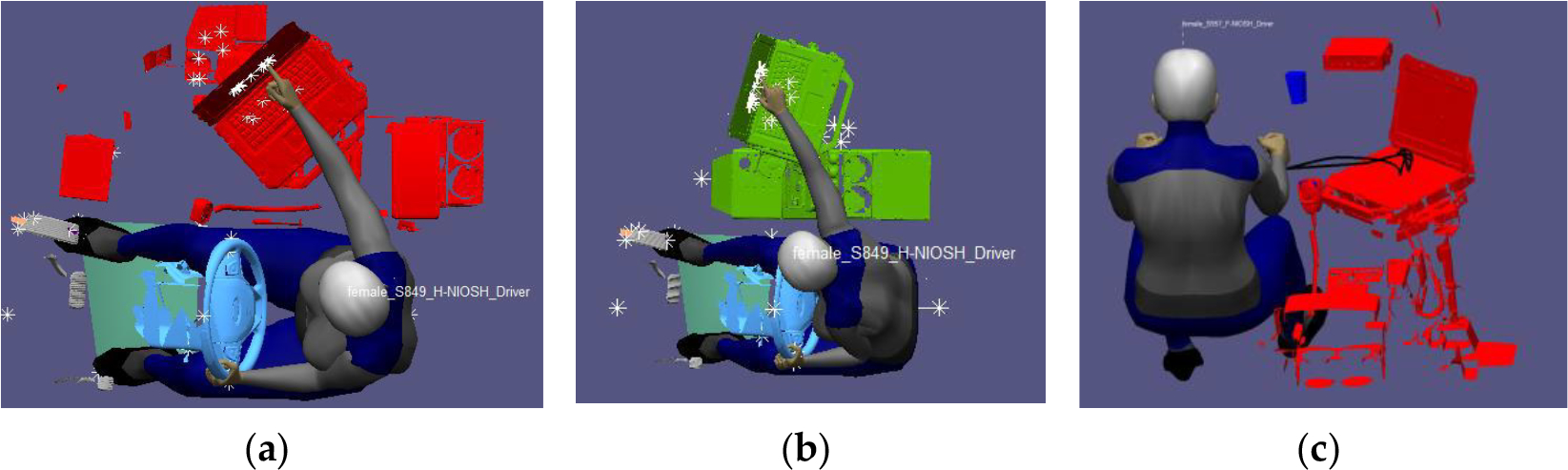
Configurations of the DVI simulated in RAMSIS: (**a**) Touch screen task on passenger-mounted MDT. (**b**) Touch screen task on center console mounted MDT. (**c**) Data entry task on the passenger seat-mounted MDT.

To ensure the DVI is properly configured, the experimental setup was examined in a simulated environment using RAMSIS. Digital human modeling tasks were performed with the 10 most representative male and 10 most representative female digital manikins selected from the 24 digital models [30]. This simulation ensured that prospective drivers would have adequate space to move within the cockpit. Figure 5 demonstrates three configurations of the DVI simulated in RAMSIS. The star symbols in this figure marked the frequent touch areas anticipated to be detected by the installed sensors. To ensure the adequacy of the configuration, each manikin was tested using 13 trials for a total of 260 modeling trials (20 manikins × 13 trials). For each individual task, the output (dependent measures) of the modeling include the time the eyesight exceeded the 35° downward angle rule, the time the eyesight confirmed the 35° downward angle rule, the number of off-road eye glances that exceeded 3 s or more, accumulative duration of off-road eye glances, and completion time (time interval between the right hand leaving the steering to the same hand returning to the same position on the steering wheel) for each required task.

The hardware, including the instrument and the sensors, of the experimental system also allowed for adjustment to accommodate different driving scenarios, ensuring optimal instrument placement and functionality. The locations of sensors and instruments were configured according to the results of the simulation in this study. In this study, two DVIs were predetermined using RAMSIS to evaluate their levels of interference and distraction score during the mission-based routes. This metric provides important information on whether the DVI is properly designed to avoid potential dangers in law enforcement vehicles.

### 3.4. Controllers and Sensors Used for Ergonomic Analysis

**Figure 6.**
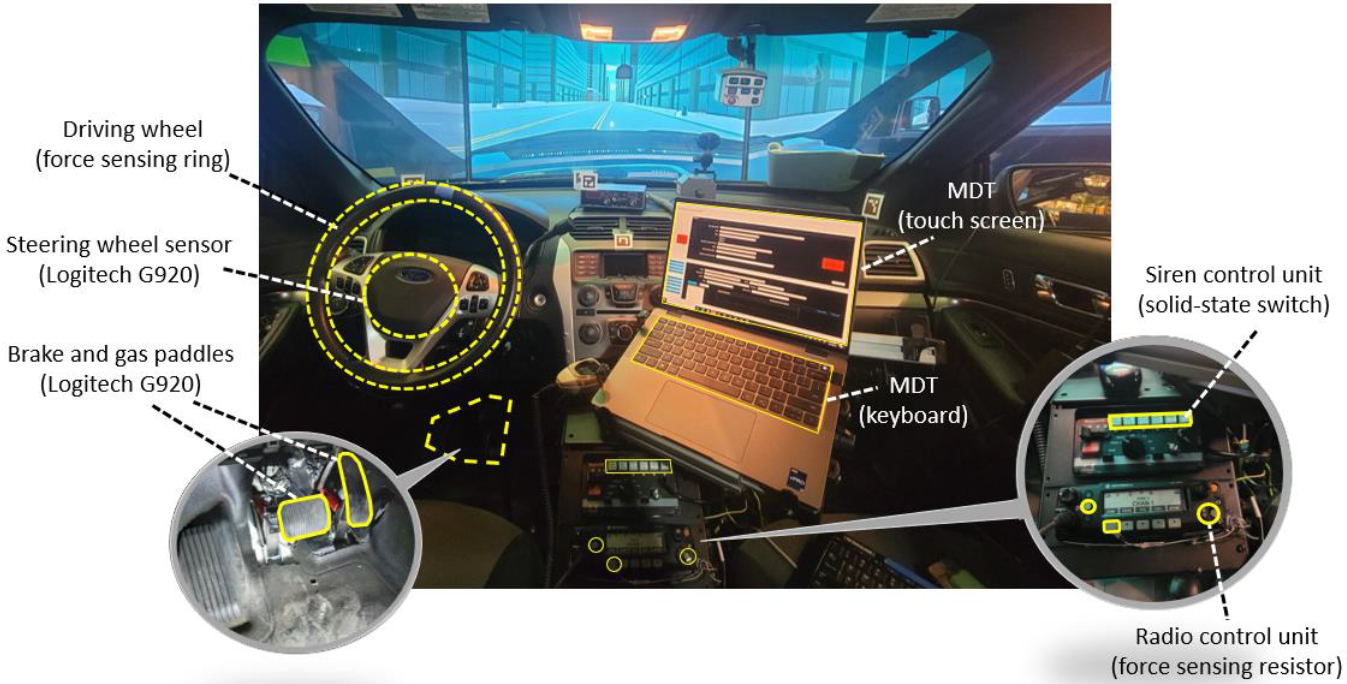
Sensor installation locations within the internal setup of the cockpit.

To enable the driver to interact with the virtual driving scenarios, the driving simulator must be equipped with the necessary controllers and sensors. These controllers include the steering wheel and paddles, which detect the driver’s control inputs and physical responses from the steering wheel, accelerator pedal, and brake pedal. Additionally, LEOs need to use various instruments to complete their assigned missions, such as the MDT, siren control unit, and radio control unit. To detect the interaction between the driver and these instruments, sensors were attached to these devices without further distracting the driver’s attention. Figure 6 illustrates the locations where these sensors and controllers were installed.

#### 3.4.1. Controllers

**Figure 7.**
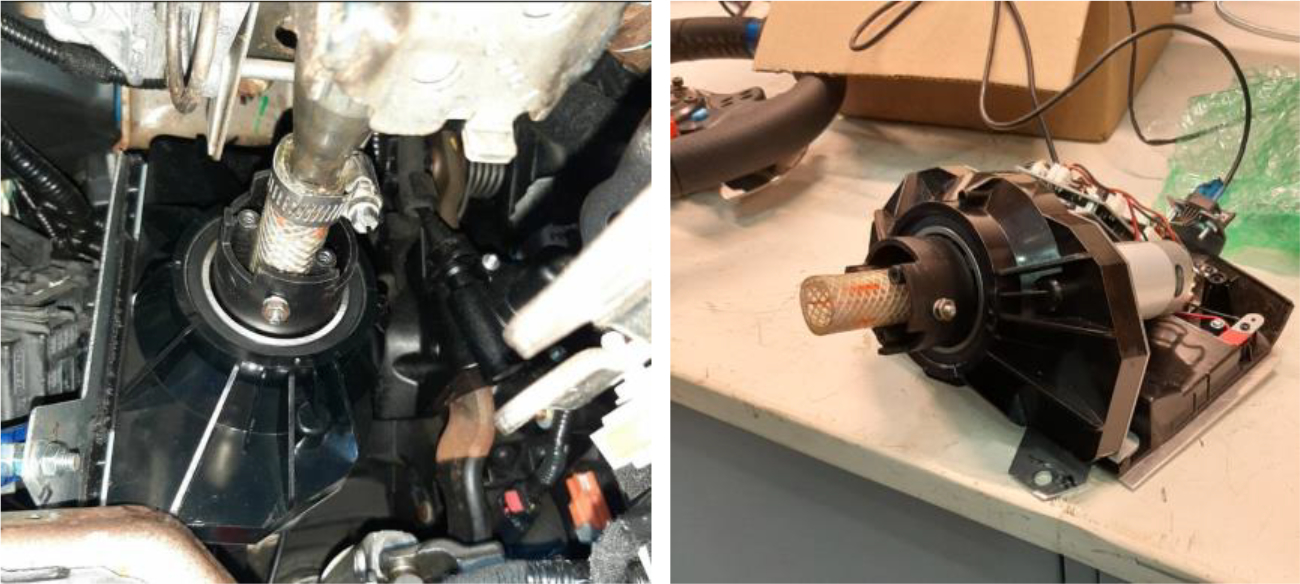
Integration and modification of the steering wheel with the Logitech G29 gaming racing wheel.

**Figure 8.**
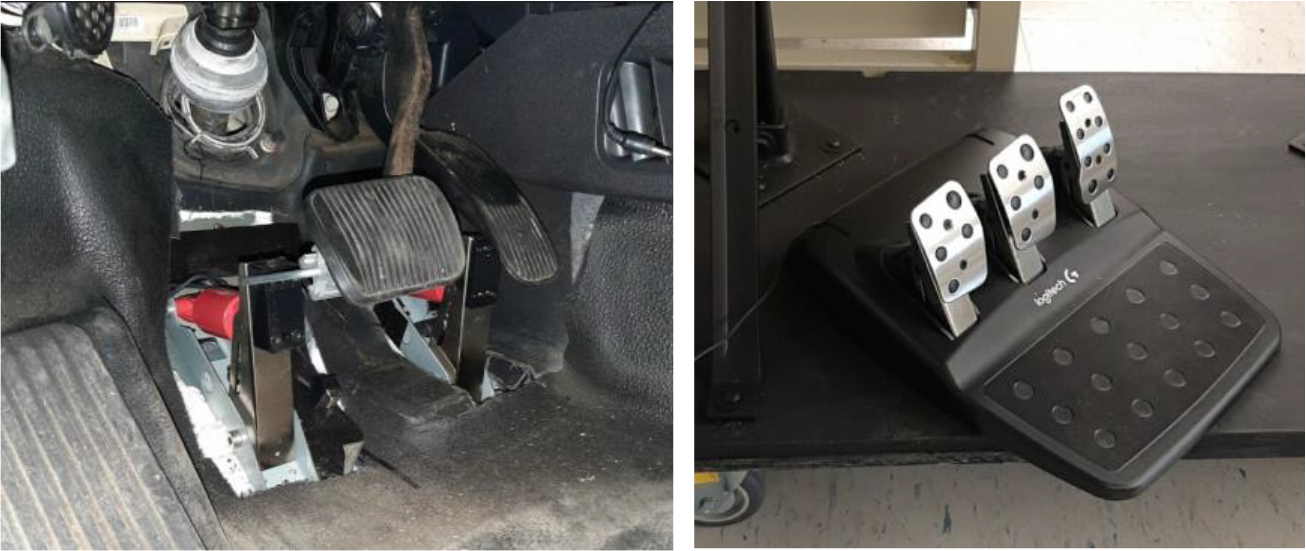
Integration and modification of the pedals with the Logitech G29 gaming controller.

For the inputs from the steering wheel and pedals, the experimental setup utilized a USB Logitech G29 driving force racing wheel and floor pedals. Instead of using the original mechanism of this gaming steering wheel directly, it was disassembled, and the encoder from a gaming steering wheel was integrated with the physical steering wheel of the Ford Police Interceptor Utility. This modification allowed the inputs from the physical steering wheel to be collected by the data acquisition system. For the acceleration and brake pedals, customized mechanisms were designed and installed to capture the inputs from the original pedals and transfer them to gaming pedals. This setup ensured that the physical inputs from both the steering wheel and the pedals could be collected via a USB connection. In this study, the buttons on the gaming controllers were not utilized. Figures 7 and 8 illustrate the designed mechanisms installed on both the steering wheel and the pedals.

#### 3.4.2. Input Device

For the MDT, a Dell Latitude 7440 laptop with a touch screen was used to emulate the MDT device. A custom program was developed to simulate the input interface, allowing the driver to manually enter data via either the touch screen or the keyboard. The location of the installation of this MDT is shown in Figure 6. Information from the MDT was not sent to the data acquisition system; instead, interactions between the driver and MDT were recorded by the event logger. In addition to its primary function, the MDT can also emulate the display of navigation information. This includes routes to mission destinations and target locations received from the control center.

#### 3.4.3. Sensors

Touch sensors were installed on the siren control unit and the radio control unit to monitor use without further distracting the driver. These control units might not be connected to the real law enforcement equipment as they were removed from this experimental platform. For example, the siren control unit was used solely for assessing the driver’s behavior, even though the physical sirens were removed.

Force Sensing Resistors (FSRs) were specifically chosen for their thin and flexible design, typically less than 0.5 mm thick, making them ideal for applications with limited space. FSRs measure pressure or force applied to their surface through a conductive polymer that changes its electrical resistance in response to mechanical pressure. When pressure is applied, conductive particles within the polymer matrix move closer together, allowing more current to pass through and lower resistance. This change in resistance is measured by interfacing the FSR with an electronic circuit, which translates the varying resistance into a measurable force output.

The primary goal of the FSR attached to the radio control unit was not to quantify the exact forces applied to the buttons but to determine whether the driver pressed the designated button. By setting a threshold for the measured signals, the data acquisition system could instantly ascertain whether the driver activated specific functions on the instruments. This setup ensured that the FSRs provided reliable input without adding to the driver’s distraction.

**Figure 9.**
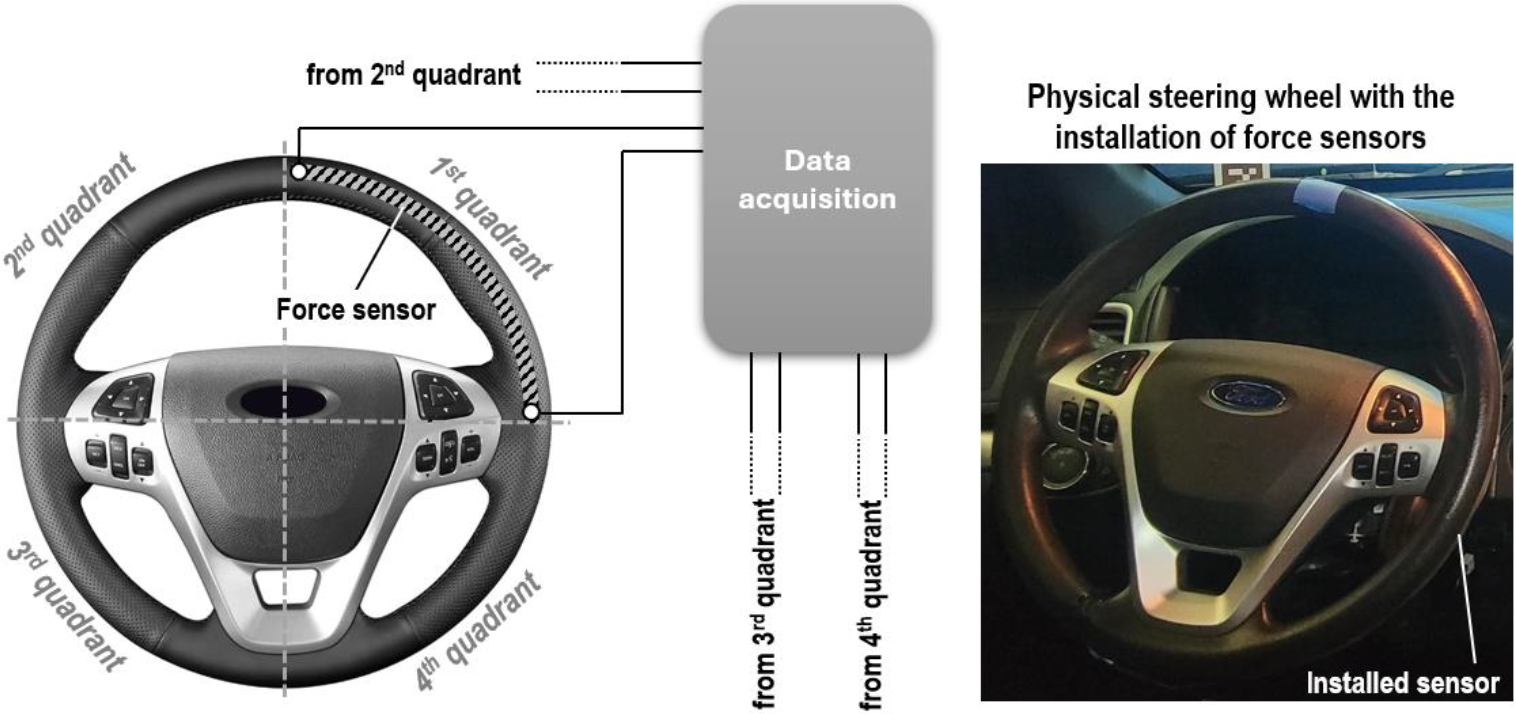
Installation of force sensors on the steering wheel.

FSRs were also installed on the steering wheel to detect which parts of the steering wheel were being held by the driver to monitor changes in driving patterns. To record the driver’s grip on the steering wheel, it was divided into four quadrants, with a force sensor installed in each quadrant. Figure 9 illustrates the installation of the FSRs on the steering wheel. This configuration enables the data acquisition system to accurately identify which parts of the steering wheel were being held by the driver and to effectively distinguish the force applied by each hand. This setup not only tracked the driver’s grip but also provided insights into hand positioning and driving behavior, contributing to a comprehensive understanding of the driver’s interaction with the vehicle. The recorded FSR signals were converted into the driver’s grip and recorded by the event logger for analysis.

In addition to the added sensors, some of the circuits in the siren control unit were also modified to capture the driver’s responses. These modifications include the buttons and knobs on the siren control panel. By installing solid-state switches, the status of these buttons could be directly measured by the data acquisition system. This adjustment ensured that all interactions with the siren control unit were accurately recorded, providing comprehensive data on the driver’s actions.

### 3.5. Data Acquisition Subsystem

The data acquisition system primarily captured the driver’s responses while navigating designated scenarios. These responses were collected from the steering wheel, pedals, siren and radio control units, and eye movements. Corresponding sensors and the dedicated data acquisition system gathered this information and fed the processed data to the physics engine. This setup enabled the driver to interact with the virtual environment in a way that ensured that their driving behavior in the virtual world accurately reflected their interactions with the physical environment.

In this study, the Visualization Workstation, a Windows 10-based computer, served as the data acquisition console. All sensors attached to the siren and radio control units, as well as the force sensor attached to the steering wheel, were connected to a National Instrument cDAQ-9174 console equipped with the necessary DAQ modules. Data from these sensors were sent to a script developed in MATLAB on the Windows system. Signals collected from Logitech G29 controllers were transmitted directly to the Windows system via a USB connection. Some of the raw data collected from sensors and controllers were filtered or converted into specified driver responses and logged by the event logger for analysis.

### 3.6. Eye-Tracking Device

**Figure 10.**
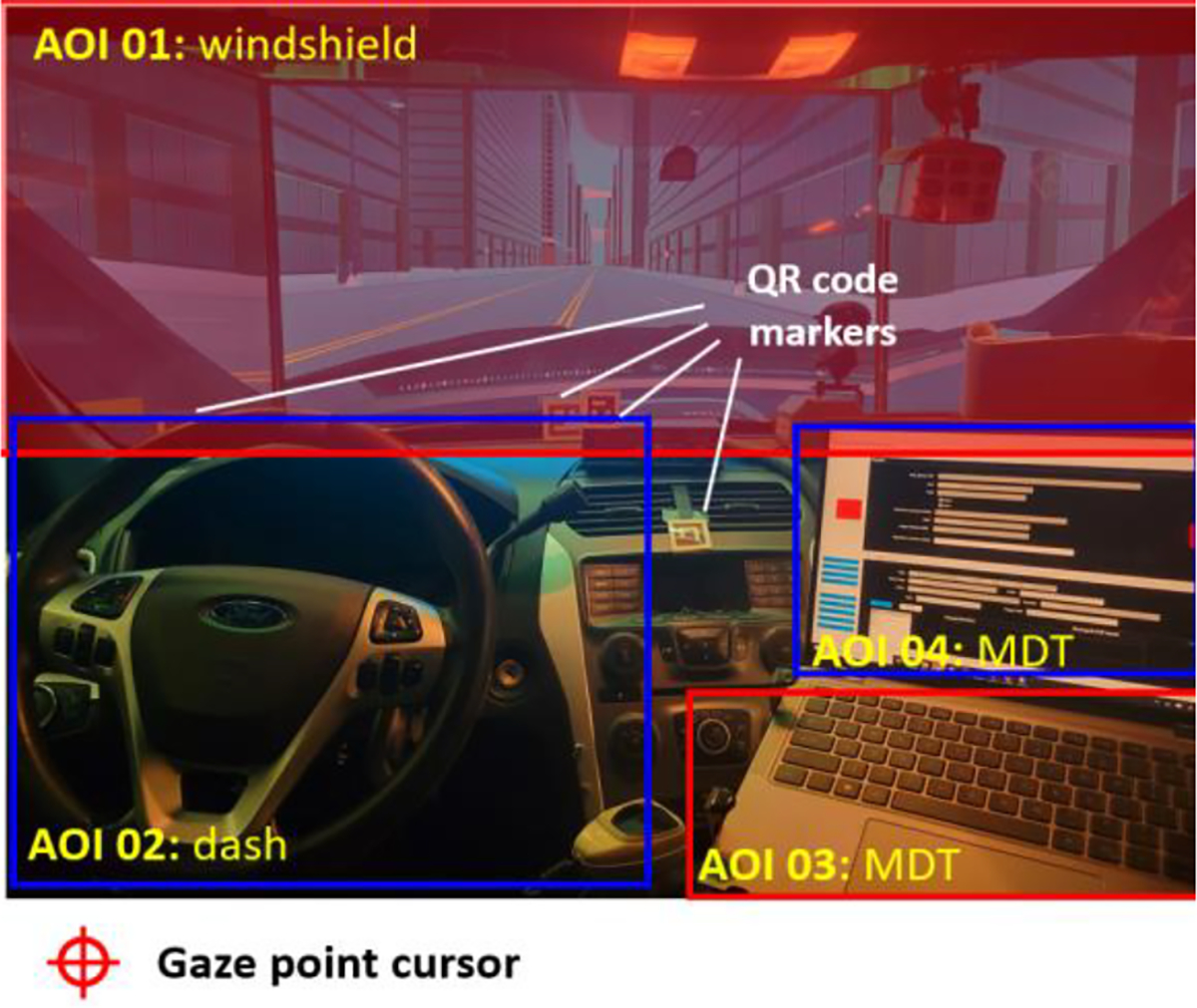
Three defined areas of interest (i.e., AOI 01, 02, 03, and 04) and QR code markers used for the eye-tracking glasses in the experimental driving simulator platform.

The eye-tracking device used in the experimental system was a separate setup that recorded eye movements independently. The recorded information needed to be post-processed to align with the durations of corresponding events logged by the event logger. The physical eye-tracking device used in this study was Ergoneer’s Dikablis PRO glasses, which tracked the movements of both eyes. The location where the eyes are looking is called the gaze point, which is displayed as a cursor on the vendor’s software package, D-Lab 3.72 (E). The QR code markers were used as reference points in the experimental environment to account for head movements. The derived location of the gaze point was then integrated with the images recorded by the scene camera to determine where the driver was focusing. To correctly record these gaze point locations, areas of interest (AOIs) were defined according to the driving scenes designed. Figure 10 demonstrates the defined AOIs and the gaze point captured by the scene camera integrated into the glasses.

## 4. Software Developed for the Experimental Platform

### 4.1. Augment Reality of the Driving Scenes

**Figure 11.**
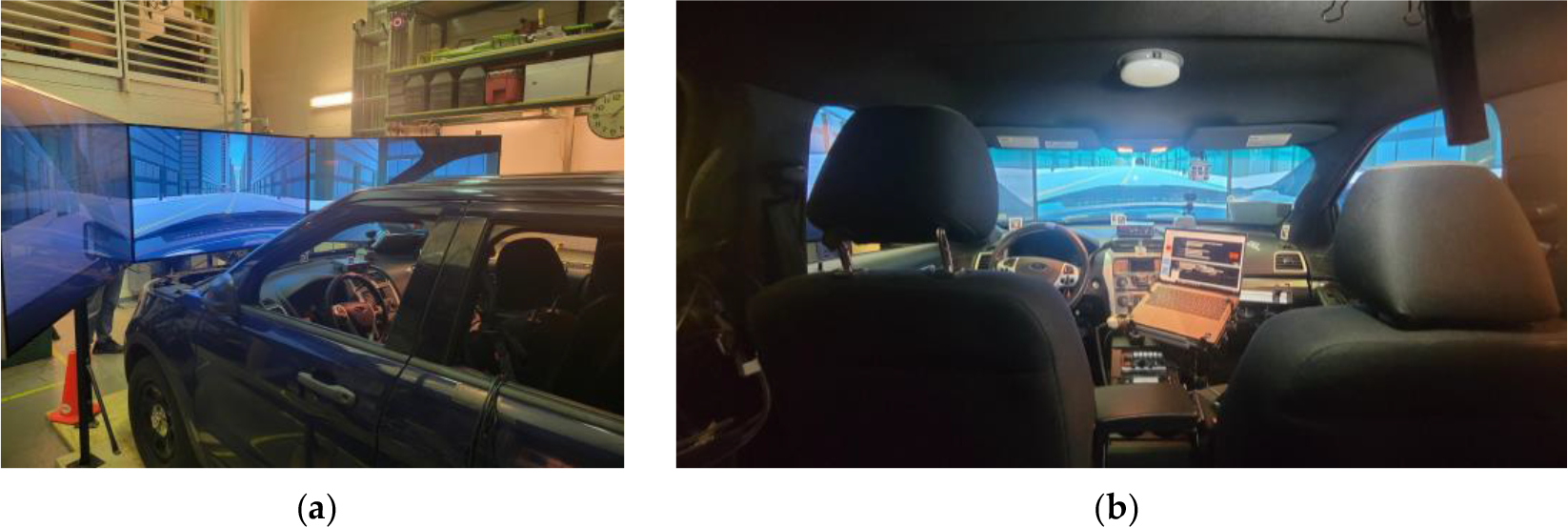
(**a**) External view of the experimental vehicle with the three flat panel displays. (**b**) Internal view of the driving scene.

**Figure 12.**
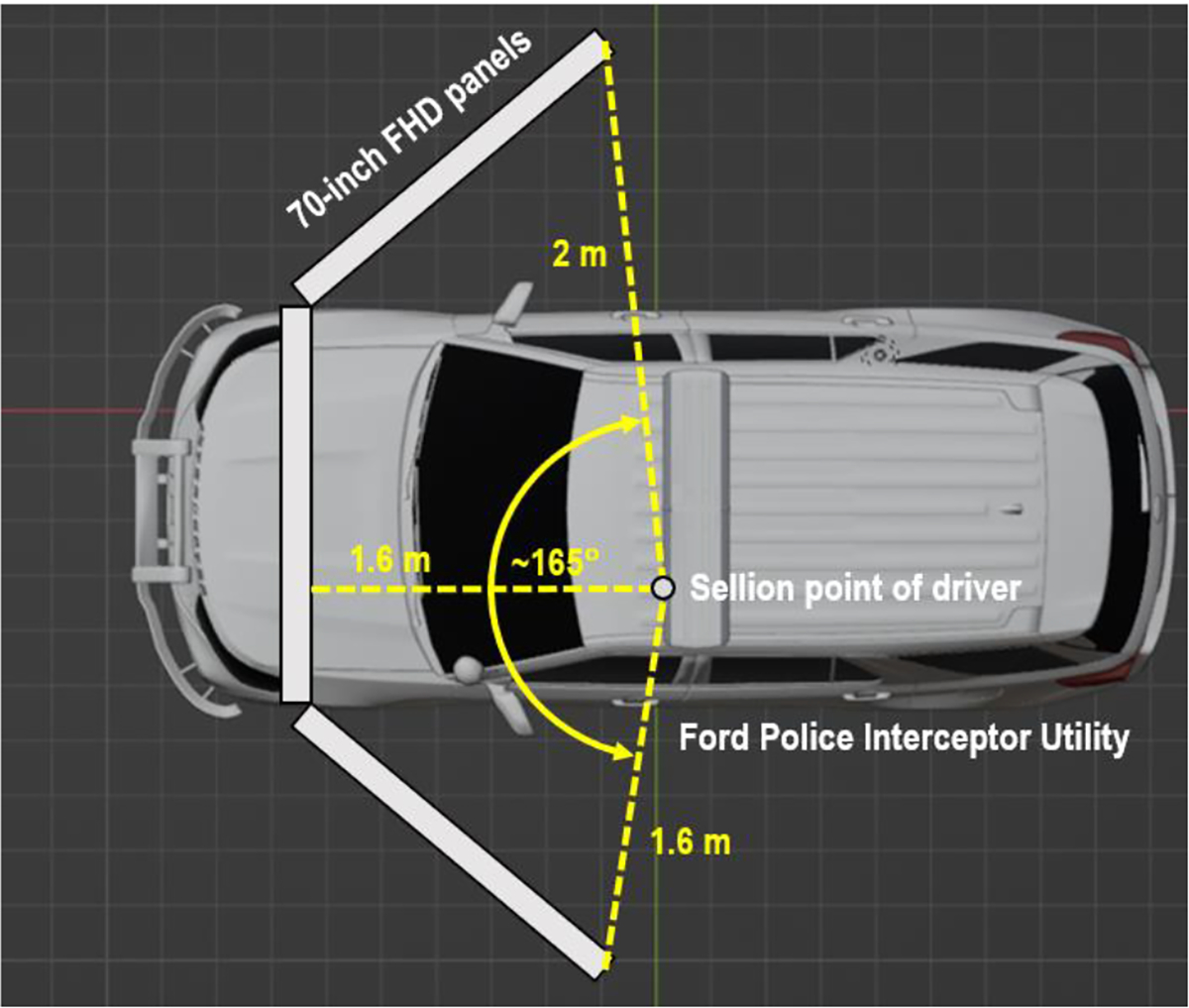
Field of view from the driver’s sellion point in the cockpit.

To create the augmented reality environment, the vehicle was positioned in a lab setting with three 70-inch full high-resolution flat panels placed in front of it. Figure 11 illustrates the integration of the virtual environment with the Ford Police Interceptor Utility. The size of the screens is large enough to cover the view of the windshield from the driver’s vantage point. The screens displayed the animations of the designed driving scenario, providing ~165° field of view from the driver’s sellion point in the cockpit, as shown in Figure 12. The dynamics of the environment, including the interacting vehicles, road conditions, and surrounding objects, were simulated using Project Chrono. After emulating the physical dynamics, the graphical display was implemented and animated using Unity to render the surrounding objects.

### 4.2. Project Chrono

To emulate the driving scenarios, the physics engine provided by Project Chrono was adopted in this experimental setup. Chrono is a physics-based modeling and simulation infrastructure based on a platform-independent, open-source design implemented in C++. The library can be embedded in software to simulate a variety of complex systems. The default 3D visualization engine of Chrono is the Irrlicht library. In developing our driving simulator, Chrono was utilized to emulate the dynamics of the environmental conditions, including interacting vehicles, road conditions, and surrounding objects.

After compiling in the Windows environment on the Chrono Workstation, the simulation scene using Chrono can directly interact with inputs from the driver, with control commands collected from the Logitech G29 via USB connection. The computer for this workstation is equipped with an i9–10900X processor, 32 GB of memory, and an NVIDIA RTX 3060 graphics card, which accelerates the calculations of vehicle dynamics. By incorporating Chrono, the physical behavior of the vehicle and its environment can be accurately simulated. To enhance the realism of the driving simulator, a sophisticated graphic rendering engine was integrated and animated using Unity, seamlessly working with the Chrono-based simulations to render the surrounding objects and create a realistic driving experience. This combination allowed this study to create a comprehensive and immersive augmented reality environment for the experimental setup.

**Figure 13.**
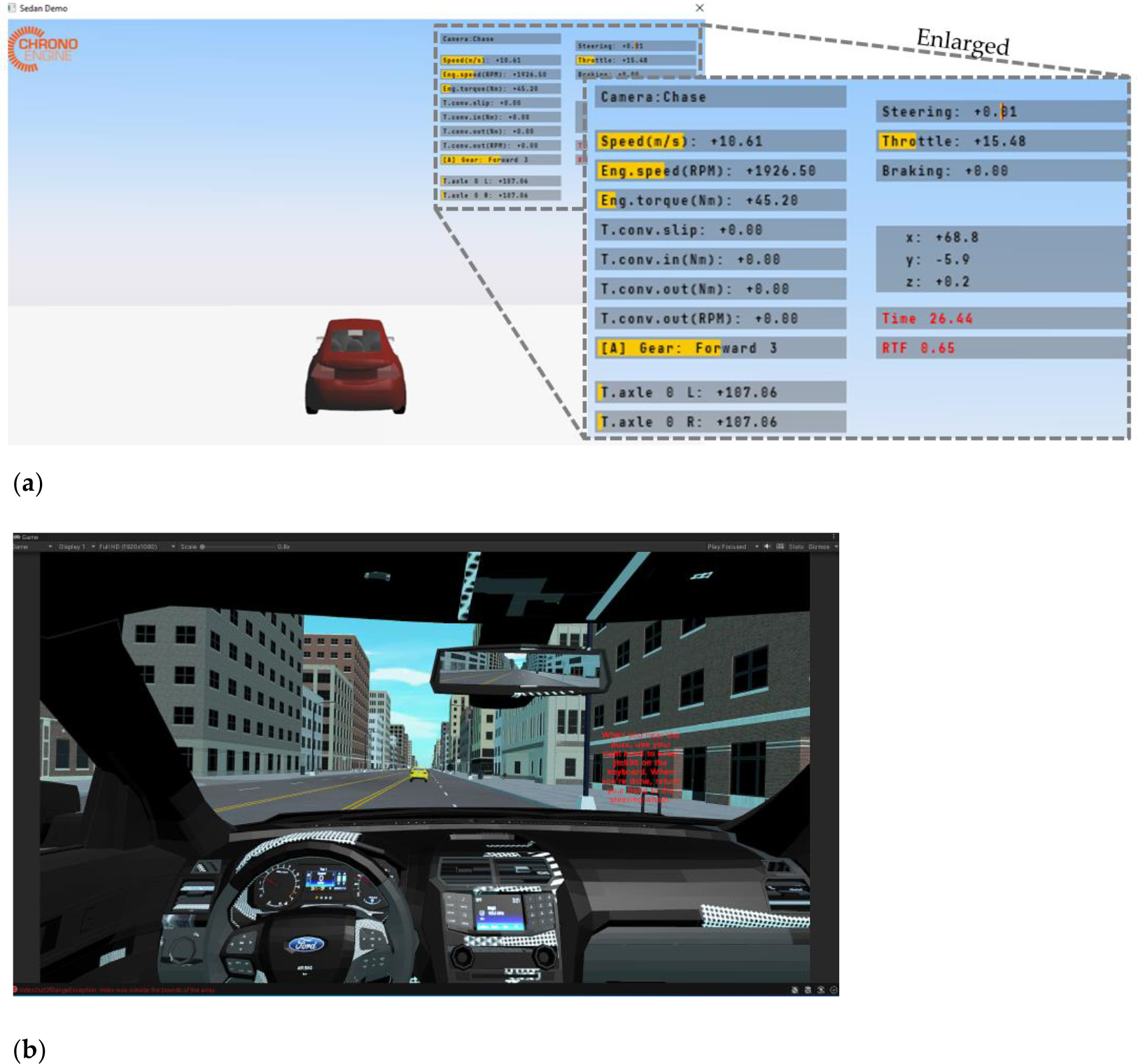
(**a**) Rendered animations on the Chrono Workstation using the Irrlicht library. (**b**) Rendered animations on the Visualization Workstation using Unity.

To implement high-quality animated driving scenes without data acquisition interference or potential latency from intensive calculations, a dedicated Visualization Workstation equipped with Unity featuring an i9–13900 processor, 64 GB of memory, and an NVIDIA RTX A5000 graphics card handled the animation. The data derived from the Chrono Workstation was transmitted via a UDP connection to the Unity-based scenario on the Visualization Workstation for animation. Figure 13 compares the rendered scenes on the Chrono Workstation and on the Visualization Workstation. It is noted that the viewing angles are different because the viewing camera on the Visualization Workstation has been moved to the driver’s seat for a more immersive simulation experience in this figure. Additionally, the viewing camera was adjusted to a position closer to the windshield to provide a view that closely resembles the driving experience in the physical vehicle on the driving simulator platform.

## 5. Ergonomic Considerations Using a Driving Simulator

### 5.1. Compensating Method for Different Users

**Figure 14.**
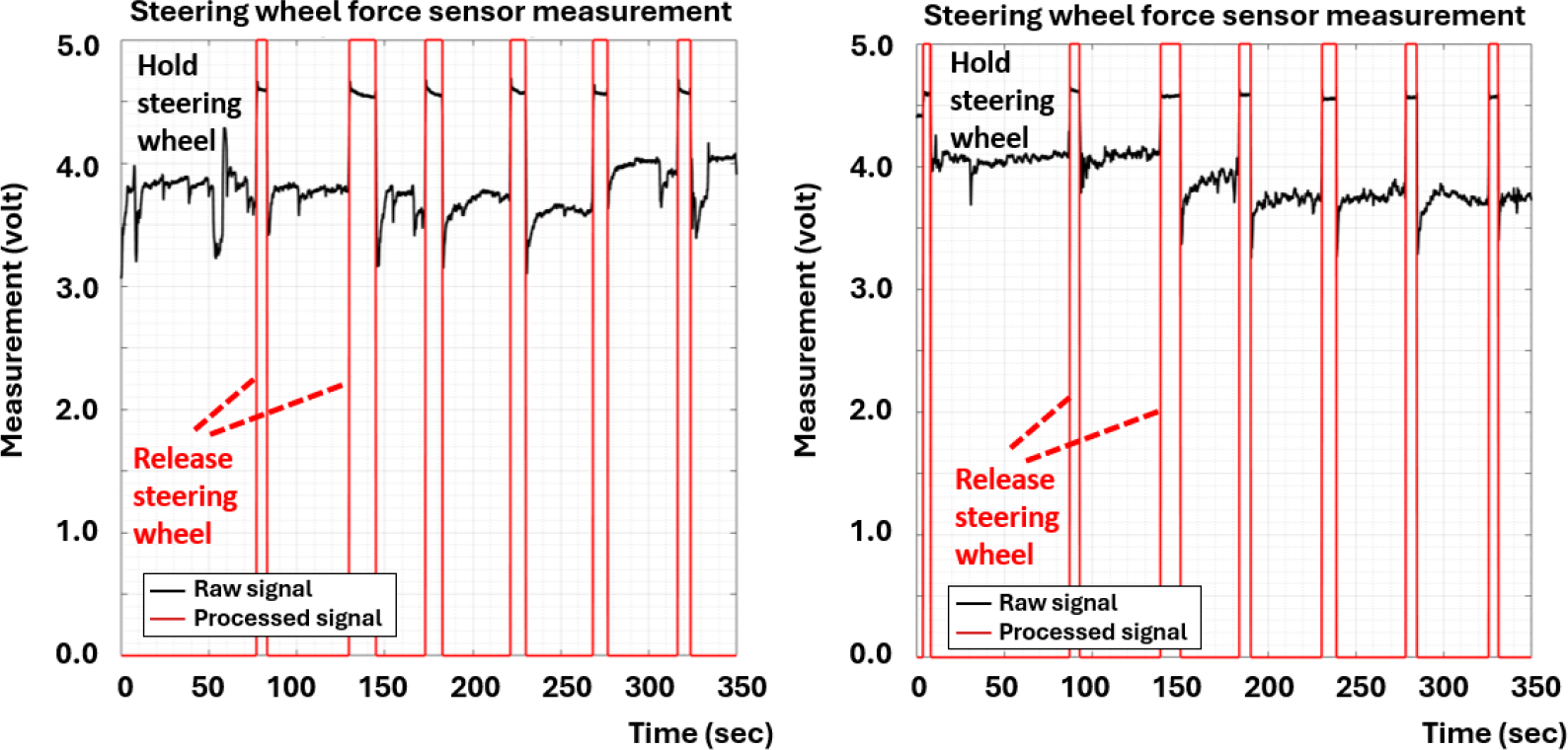
Comparison of the holding steering wheel grip pattern between two drivers.

Before analyzing the measured signals from the sensors and inputs from the controllers, some of these raw signals must be converted into other formats for behavior analysis. Specifically, the measured signals can exhibit different patterns due to factors such as gender, age, or health conditions. Converting these signals into corresponding status indicators of triggered events simplifies future analysis. Figure 14 demonstrates the raw signals recorded from the FSRs attached to the steering wheel, acquired from two distinct drivers. It was evident that the force measurements of individual drivers holding the steering wheel can vary significantly, making it challenging to develop a universal analog function to detect whether a driver is holding or releasing the steering wheel. While the recorded signals alone can be challenging to interpret in terms of driver behavior, the status becomes much clearer once these raw measurements are converted into corresponding discrete events. In particular, applying the Kalman filter to the force acquisition measurements can significantly enhance the readability of the raw signals, making them much easier to interpret and assess.

The conversion from raw measurements to event status was not limited to just the FSRs attached to the radio control unit and the steering wheel; it was also applied to the solid-state switches of the siren control unit. These events are then recorded by the event logger for analysis of distraction levels and other driver-related properties.

### 5.2. Ergonomic Considerations and Distraction Index

In a driving simulator, evaluating and quantifying driver responses is essential to determine the distraction level. Distraction can significantly increase response time by diverting the driver’s attention away from the primary driving task, causing slower cognitive processing and reaction. For example, when a driver is interacting with onboard devices or is visually or cognitively distracted, their ability to promptly respond to sudden changes in the driving environment, such as a pedestrian crossing the street or a vehicle stopping abruptly, can be impaired. Key considerations to measure include attention time, response time, glance duration, hand movements, and interaction with onboard devices. The following list outlines the factors used in this study, and Table 1 explains how the ranges for these factors were determined.

- Attention Time (AT): The percentage of time the driver’s eyes are on the road is crucial to measure as it directly impacts situational awareness and reaction time to unexpected events. Advanced eye-tracking systems monitor where the driver is looking at any given moment, providing data on how much time is spent focusing on the road versus being distracted by in-vehicle instruments.
- Response Time (RT): Response time is the duration it takes for a driver to perceive a stimulus and initiate an appropriate action, such as braking or steering. Delayed response time indicates a lag due to distractions, increasing the risk of accidents. Response times vary with the type of incident and evasive maneuver. According to Dewar and Olson [33], most drivers (approximately 85 to 95%) can respond within about 1.5 s to a clear stimulus in a straightforward situation. However, distractions and eyes-off-road can further delay response times by 16% and 29%, respectively [34].
- Glance Duration (GD): The maximum duration of glances away from the road towards various instruments in the vehicle is measured to identify lapses in attention. By quantifying these glances, researchers can identify which instruments are more likely to cause distraction and potentially redesign their interfaces or placement within the vehicle.
- Hand Movement (HM): Measuring the time it takes for a driver to move their hand from the steering wheel to another control unit, like the MDT or radio, and back provides insight into the physical demands placed on the driver. These data help determine if the layout of controls contributes to distraction by requiring excessive or awkward hand movements.
- Interaction Frequency with Onboard Devices (IF): The frequency and complexity of interactions with onboard devices, such as adjusting the siren, using the radio, or inputting data into the MDT, were also evaluated. Force sensing resistors (FSRs) and touch sensors capture how often and how intensively drivers interact with these controls. Frequent and complex interactions can indicate higher distraction levels, suggesting a need for interface improvements or alternative methods of operation, such as voice commands.

By considering all these metrics, researchers created a comprehensive profile of driver behavior and distraction levels. With these metrics, a composite distraction index (DI) prediction model was used. However, the DI does not exhibit a linear relationship with any single metric. Instead, it is influenced by various combinations of metrics. Therefore, a supervised machine learning model was appropriate for determining whether the driver is distracted or focused on the road. However, the DI value is a binary indicator (true or false) determined by the prediction model based on the driver’s response to encountered events while driving. To accurately assess the level of distraction for the mission, the frequency or duration of the DI was further analyzed while the driver was on the mission-based route.

To develop such a prediction model using machine learning, we normalized the input features. In this study, the data on driver responses were emulated, resulting in 200 sets of data generated from preliminary studies according to personal communication (Cheng, M.; Guan, J. June 10, 2024) [35]. The generation of the driver response data was based on known properties and assumptions. These emulated data were divided into 60% for training, 20% for validation, and 20% for testing. A Gradient Boosting Regressor was selected for the model due to its capability to handle diverse data types and its robustness against overfitting. The model’s performance was evaluated using the Mean Squared Error (MSE) metric. Additionally, the importance of each feature was analyzed to understand the impact on the distraction index.

To build the model, the baseline information of the factors needed to label the distraction index in the datasets used for training was required. In this study, the baseline data were obtained from the literature. One study investigated the effects of a driver monitoring system that triggered attention warnings when distraction was detected [36]. According to the Euro NCAP protocol [37], distraction can be defined as either a long glance away from the forward roadway (≥3 s) or visual attention time sharing (>10 cumulative s within a 30 s interval). Since each test lasted longer than 30 s, the total glance away time was converted to the percentage of attention time based on this definition. For this study, an attention time (AT) period of less than 70% was marked as distracted. Regarding the glance duration (GD), it was defined as the longest duration that the driver glanced away from the forward roadway. If this duration exceeded 3 s, the data were marked as distracted. For the response time (RT), a 95% threshold of 1.5 s was used to determine whether the driver was distracted. With the increasing use of automation mechanisms in modern vehicles, hands-off-wheel time has risen and correlates with eyes-off-road time. The number of hand movements varied from task to task. In a test where the driver glanced at the monitor to read and write text on the phone, the duration of glances was 2.14 s (with a standard deviation of 2.06 s) and 1.22 s (with a standard deviation of 0.79 s) [38]. Therefore, for a task that required the driver to input six characters (three letters and three numbers), an average accumulated duration of 3.36 s was anticipated. Although shorter hand movement (HM) duration might be preferred, a threshold of 3.3 s was used to label distraction.

### 5.3. Development of Machine Learning Model for Distraction Index

The machine learning model developed in this study aimed to evaluate DI for LEO’s driving reaction during mission-based routes. The Gradient Boosting Regressor (GBR) algorithm was selected for its robust performance in regression tasks, particularly when handling complex datasets and non-linear relationships [39,40].

The modeling process began with data collection on driver’s behaviors. Sensors installed in the vehicle recorded various metrics, including eye movement, the force exerted on the steering wheel, and environmental factors such as road conditions and targeted tasks. An event logger captured instances of specific actions and reactions, such as the use of MDTs, radio communication, and siren controls. These events, along with their timestamps, provided a comprehensive dataset that combined both continuous sensor data and discrete event logs. After data collection, feature engineering was conducted to extract meaningful variables influencing driver distraction. This process included the creation of time-series features from sensor data, event frequency counts, and duration metrics. Interaction terms between different types of events and sensor readings were generated to capture more complex distraction patterns. For example, the frequency and duration of MDT usage while driving at high speeds were specifically calculated as critical indicators of cognitive overload. The collected data were labeled for training purposes, ensuring the model could learn and predict distraction levels accurately.

The GBR algorithm was employed for its ability to handle both the linear and non-linear aspects of the data effectively. It built an ensemble of decision trees, where each subsequent tree aimed to correct the errors of the previous ones and gradually improve the model’s predictions.

- Initialization: The process begins with a simple initial model, typically a mean predictor.
- Iterative Boosting: In each iteration, a new decision tree is trained on the residual errors (i.e., the difference between the predicted and actual values) from the previous model. This new tree is then added to the ensemble with a specific weight.
- Learning Rate: The learning rate is a crucial parameter that determines the contribution of each tree to the final model. A lower learning rate generally leads to better performance but requires more iterations.
- Regularization: Techniques like shrinkage (applying the learning rate) and subsampling (using a random subset of the data for each tree) were employed to prevent overfitting and enhance the model’s generalizability.

The performance of the GBR model was evaluated using cross-validation techniques to ensure its robustness and accuracy. Metrics such as Mean Squared Error (MSE) and R-squared (R^2^) were used to quantify the model’s predictive power. Grid search methods optimized hyperparameters, including tree number and depth, and learning rate.

To maintain the model’s accuracy over time for the driver’s reaction, real-time monitoring results were continuously fed back into the system. This ongoing data collection allowed for periodic retraining of the model, ensuring that it adapted to changes in driver behavior and new patterns of distraction. During retraining, each factor was reevaluated to confirm its relevance and impact, thus keeping the model aligned with the current driving conditions and officer tasks.

The final model provided insights on DI under various DVI configurations. This information helped optimize instrument placement in law enforcement vehicles, aiming to minimize distractions and enhance officer safety. By refining DVI settings, the model supported officers in maintaining focus during critical tasks, contributing to safer driving conditions and reduced accident rates.

## 6. Determination of Distraction Index

The primary advantage of a driving simulator that emulates law enforcement vehicles is its ability to help manufacturers optimize the configuration of internal instruments, thereby minimizing distractions while driving. Additionally, it can be used to test LEOs’ responses to innovative technologies, allowing potential distractions to be identified and addressed before these technologies are deployed. This section discusses various driving scenarios and presents a case study for the developed driving simulator.

### 6.1. Common Police Missions and Associated Distractions during Regular Driving

LEOs perform a variety of missions while driving, many of which involve tasks that can be distracting due to the use of special equipment. They frequently need to operate in-car computer systems, communicate via radio, and engage with the surrounding environment, all while maintaining situational awareness and control of the vehicle.

- During routine patrols, officers continuously monitor their surroundings for suspicious activity or traffic violations. This requires frequent glances at their mobile data terminals (MDTs) to check for updates or run license plate checks, which can divert their attention from the road. Additionally, communicating with dispatchers and other officers via two-way radios necessitates the use of hands-free devices, which can still be a source of distraction if officers need to manipulate the radio controls.
- Responding to emergency calls is another mission that demands high levels of attention and quick reactions. Officers must navigate through traffic at high speeds, often using sirens and light bars to signal their presence. Operating these controls while simultaneously reading navigation systems or receiving real-time updates on the MDT can lead to significant distractions.
- Surveillance operations involve following suspect vehicles discreetly while maintaining communication with other units. Officers use spotlights, dash cameras, and vehicle tracking systems during these missions. Manipulating these instruments, along with monitoring the suspect and their own driving, can be highly distracting.
- High-speed pursuits are among the most dangerous and distracting missions. Officers must maintain control of their vehicles at high speeds while coordinating with dispatch and other units. The need to constantly update their position and status using the MDT or radio, alongside operating the vehicle’s emergency systems, can severely impact their focus and reaction times.

In summary, while regular driving, LEOs engage in missions that require the use of various equipment, leading to potential distractions. The experiments designed in this study aim to emulate various law enforcement scenarios to evaluate the distractions caused by the functions that participants need to perform in the vehicle.

### 6.2. Time Sequences and Durations of Tasks in Assigned Missions

To determine the DI caused by the special equipment while driving, a series of tasks were assigned to the driver at specific moments during the test. Each task was designed to simulate real-life scenarios that LEOs might encounter. The details of these individual tasks are outlined in Table 2.

**Figure 15.**
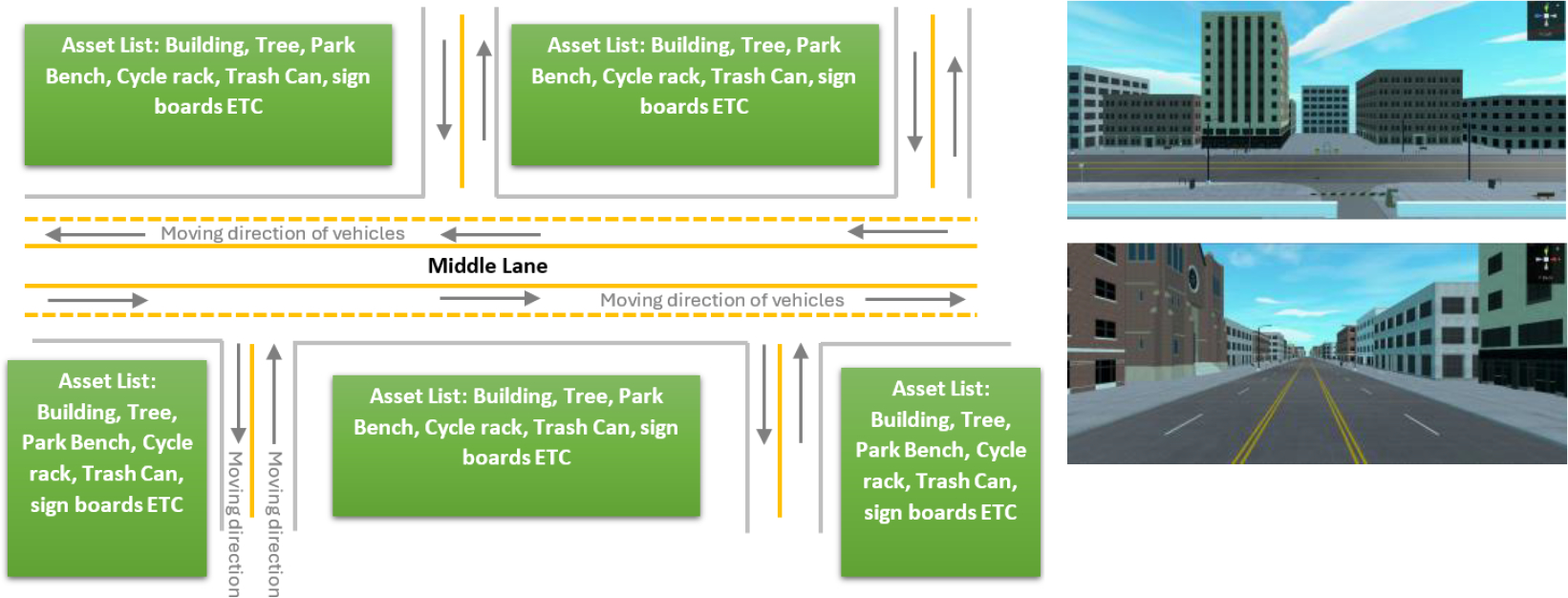
Planned driving scenario and the corresponding implementation in Unity.

The driving scenario in the test was based on a regular city scene. The virtual scene consisted of a ~160 m street designed with various buildings. Figure 15 illustrates the layout of the city scene segment and its implementation in Unity. The segment was repeated and stitched together to create a continuous route, enabling the participant to perform a 90 min driving simulation. This synthesized route included a variety of urban elements and traffic conditions to provide a realistic and comprehensive driving experience for the test.

To ensure the driving behavior in experiments can closely reflect real-world scenarios, the sequence of designated tasks was randomized. Additionally, the two different DVI settings were randomized for participants’ test drives. This randomization process was implemented to minimize the effects of anticipation, route familiarity, and learning.

The experiment consisted of three 33 min test drives for each driver under two different DVI setups. During each drive, the participant was required to complete four different tasks listed in Table 2. The sequence of the tasks performed was randomly selected for each test drive. Before each task began, the simulator system audibly presented a pre-recorded voice instruction detailing the task to be performed. Then, a buzzer was activated to prompt the participant to perform the designated task.

**Figure 16.**
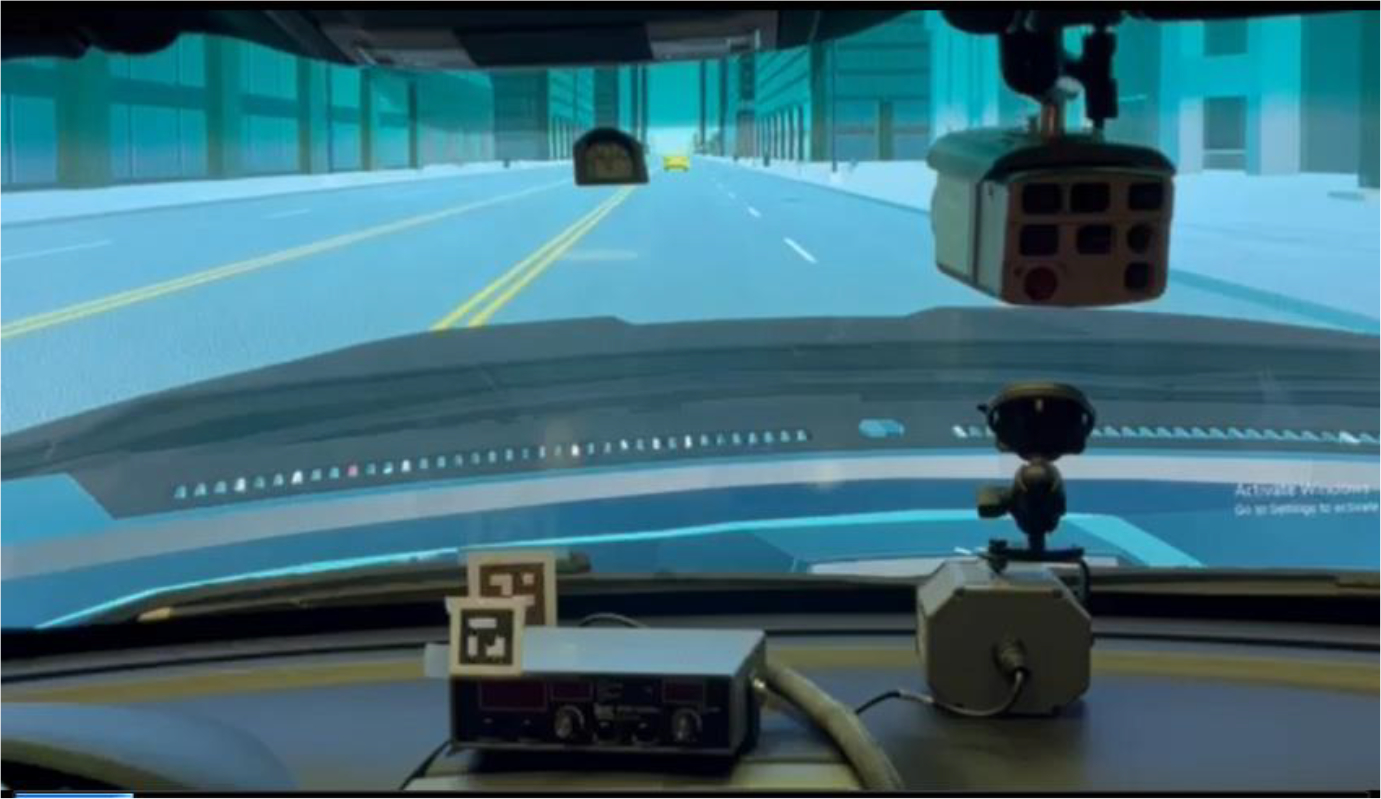
Driving scenario where the driver follows a yellow vehicle with varying speeds viewed from the cockpit.

Throughout the test drive, the participant was instructed to follow the 3 s rule [41] and keep a safe distance from a yellow vehicle positioned in front of them. This lead vehicle’s speed fluctuated between 15 and 25 miles per hour, following a sinusoidal pattern. This variation was intended to simulate realistic driving conditions and challenge the driver’s ability to focus on both the driving task and the assigned tasks simultaneously. Figure 16 demonstrates the driver’s view while following the yellow vehicle at varying speeds. In addition to maintaining a safe distance from the lead vehicle, the driver had to perform the specific tasks requested as part of the mission. These tasks were introduced at different times during the drive, ensuring that the driver had to continuously switch their attention between driving and task completion. This setup allowed researchers to evaluate the driver’s distraction levels and how effectively they could manage multiple demands on their attention.

The combination of maintaining a safe distance from the fluctuating lead vehicle and performing assigned tasks provided a comprehensive measure of the driver’s distraction levels. This experimental procedure aimed to replicate the multifaceted challenges faced by LEOs in the field, thereby providing valuable insights into the design and effectiveness of in-vehicle instruments and interfaces.

### 6.3. Assessed Ergonomic Parameters

To assess the DI caused by the assigned tasks, various parameters were quantized. With this built experimental driving simulator, the following three parameters of a driver were the major factors measured in this study: (1) the percentage of attention time the driver’s eyes on the road and on designated tasks, (2) the maximum duration of glances towards required devices, and (3) the total hand movement time required to complete the assigned task. By comparing these values across different DVI settings, an optimal DVI configuration was identified to minimize the distraction levels. Through careful evaluation and adjustment of DVI settings, a more effective system can be developed to support officers in performing their duties with greater focus.

The frequency of DI introduced in the previous section can also serve as an indicator of the distraction level. Generally, a lower frequency of DI suggests a higher level of concentration on the road, while a higher DI frequency may indicate that the driver is potentially distracted by events or other road conditions. In such cases, a warning system may be necessary to alert drivers to refocus on their driving tasks.

To evaluate whether the driver is distracted by assigned tasks, all events and the durations of the driver’s responses were recorded. Table 3 lists the sequence of the four tasks of a single test drive. Before each task started, a buzzer was activated to alert the driver to the upcoming event. The driver then needed to respond to a voice prompt and follow the instructions. All events, including the timestamps of individual events recorded by the sensors and the eye focus recorded by the eye-tracking system, were saved in the event logger. A detailed report was created after each test drive. This report can then be used to assess the DI status of the driver during the assigned mission-based route. Additionally, the report can inform improvements in task design and instrument layout to enhance safety for LEOs.

### 6.4. Prediction of Distraction

To assess the distraction index caused by the assigned tasks, various parameters can be quantized. The following three parameters were measured in this study: (1) the percentage of attention time the driver’s eyes were on the road and on designated tasks, (2) the maximum duration of glances toward required devices, and (3) the time required for hand movements from one device to another. By comparing these values between DVI settings, an optimal DVI configuration was identified to minimize distraction levels. Table 4 presents five samples from the 200 sets of emulated data used for training the prediction model of driver response. These emulated data have been labeled as true or false according to the criteria specified in the previous section based on a baseline test and the values found in the literature [36–39]. These data were used to model the prediction of distraction index.

The synthesized prediction model was utilized to assess the driver’s distraction index. Algorithm 1 outlines the procedure for determining the DI and refining the model based on ongoing recorded data. The prediction results based on the derived machine learning model M provided insights into the drivers’ distraction levels during simulated driving scenarios. Through the utilization of various features such as response time, interaction frequency with onboard devices, and other relevant metrics, the model effectively predicts DI for each emulated driver.

**Algorithm 1.** Prediction of distraction with the developed model

**Input**: Real-time sensor data: Attention Time (AT) Response Time (RT) Glance Duration (GD) Hand Movement (HM) Interaction Frequency (IF)
**Output**: Distraction Index (DI)
**Initialization**: • Train the Gradient Boosting Regressor model M using historical data. • Set up real-time data streams for AT, RT, GD, HM, and IF.
**Procedure**: Data Preprocessing: • Normalize the real-time input data to match the scale of the training data; • Handle missing values through imputation or exclusion; • Extract additional temporal features if necessary. Feature Vector Construction: • Construct the feature vector $X_{t}\;=[AT_{t},RT_{t},GD_{t},HM_{t},IF_{t}]$ at each time step t; Prediction: • At each time step t: ⚬ Predict the Distraction Index using the trained model: $DI_{t}\;=\;M.predict(X_{t})$ • Real-Time Monitoring and Feedback: • Continuously monitor the predicted $DI_{t};$ • If $DI_{t}$ exceeds a predefined threshold τ: ⚬ Trigger a warning system to alert the driver to focus on driving tasks. Continuous Learning: • Periodically collect new labeled data from the real-time system; • Retrain the model Mwith new data to improve accuracy and adapt to new driver behavior patterns. **End Procedure**

The model’s performance metrics, such as accuracy, precision, recall, and F1 score, provide a comprehensive evaluation of its predictive power. These metrics offer insights into the model’s ability to correctly classify distracted and non-distracted driving instances, thus aiding in assessing its overall effectiveness. By understanding which features have the greatest impact on the DI, interventions and countermeasures can be developed to mitigate distractions and enhance road safety. With the prediction model processed using an i7–1365U processor, the required time to determine whether the driver is distracted by surrounding road conditions or designated tasks is approximately 0.17 s. This quick response time of the prediction model is efficient and feasible for generating a warning signal to prompt the driver to refocus on the road, provided that all required factors are measured in real time. The index can then be used to either temporarily elevate the level of vehicle autonomy or generate a warning signal, ensuring the vehicle can be controlled properly and safely. If the DI persists beyond a threshold interval τ, the warning system should be activated to alert the driver.

To enhance the accuracy and reliability of the prediction model, real-time monitoring results can be collected as new input data to retrain the model. This enables the prediction model to be updated based on the current driver’s behavior, capturing changes in the driver’s behavior over time.

## 7. Advantages and Disadvantages of the Proposed Platform

While this study provided a feasible tool to determine the DI and assess distractions of an LEO on a mission-based route, the proposed platform has several limitations that may require improvement.

Firstly, the AOIs need to be dynamically adjusted based on the orientation of the driver’s head. The human field of view can concentrate on only a very narrow area, approximately 20° [42]. Therefore, the AOIs defined in the software might need to be adjusted according to the driver’s pose and the ongoing events. Ongoing adjustment requires additional studies to refine the accuracy of AOI adjustments.

Secondly, the eye-tracking system used in this study was lab-level equipment, which is not available in standard police cruisers. This study required a significant preparation time to ensure the precise functionality of the instruments, making it impractical for use by LEOs during mission-based routes. To address this limitation, alternative methods could be explored. For instance, combining data from cameras equipped in the vehicle with deep learning techniques could derive the gaze point and provide a more feasible way to evaluate driver distraction [43,44] and to identify which features of the equipment attract the most attention.

Providing excessive information can exacerbate driver distraction, diverting attention from monitoring road conditions and potentially increasing the risk of hazardous situations. Additional research is needed to design non-distracting warnings within the DVI [45]. While the study presents a solid foundation for assessing driver distraction among LEOs, there is room for improvement. Addressing the limitations related to AOI adjustments, the practicality of eye-tracking equipment, and exploring alternative methods, such as deep learning-assisted camera systems, can enhance the platform’s effectiveness and applicability.

Effective warnings should prompt the driver to refocus on driving without causing additional distractions. Design considerations of these warnings should incorporate visual and auditory cues that are easily noticeable yet unobtrusive. For example, subtle visual alerts, such as changes in dashboard lighting or small, targeted notifications, can prompt the driver to pay attention without overwhelming them. Additionally, gentle auditory alerts, like soft chimes, can serve as reminders without being startling.

The distribution and frequencies of DI values across categories for various tasks can be used to assess the adequacy of the DVI layout as well. If drivers are distracted during specific tasks due to the cognitive requirements associated with completing them, the layout of the involved instruments should be reviewed for a safer design. Ensuring the optimal placement of these instruments is important to minimize potential distractions and enhance the safety of LEOs, especially during high-stress situations and emergency responses. A poorly designed DVI can lead to operational errors, delayed reactions, and increased risk of incidents, thereby endangering both officer safety and public safety [46].

To quantify the effect of different DVI settings, a multivariate analysis of variance, or MANOVA, can be applied to compare the Existent and Modified Configurations for task completion time and eyes-off-road time. If the overall F-statistic is significant for task completion time, univariate analyses will be conducted to compare the configurations on task completion time for each of the four tasks individually. Similarly, if the overall F-statistic is significant for eyes-off-road time, univariate analyses can then be conducted to compare the two configurations for eyes-off-road time for each of the four tasks individually. These baseline trials have been designed to provide a heuristic value of eyes-off-road time during normal driving only.

## 8. Conclusions

In conclusion, this study sought to address the critical issue of distraction among LEOs while driving, highlighting potential risks associated with the increasing presence of in-vehicle instruments and devices. Through a review of existing literature and empirical data, this study underscores the need to understand and mitigate driving distractions in law enforcement contexts. The establishment of an integrated driving simulator platform tailored specifically for law enforcement scenarios represents an essential step toward addressing these challenges. By accurately capturing and analyzing various aspects of driver behavior, such as responses to specific stimuli and external distractions, this experimental platform enables a comprehensive assessment of driving performance and the effectiveness of different driver–vehicle interface (DVI) designs.

The development of this simulator provides invaluable insights for research and development. By replicating diverse driving scenarios within a controlled setting, the risks associated with the newly designed DVI incorporating advanced technologies can be assessed based on LEOs’ reactions in a controlled laboratory environment. This approach allows for thorough evaluation before implementing these enhancements in real-world vehicles, thereby improving the safety and efficiency of law enforcement operations. Overall, this study contributes to the body of knowledge on driving safety and offers practical recommendations for improving the operational safety of LEOs. By addressing the nuanced challenges of distraction in law enforcement driving, this research aims to foster safer roadways and more effective law enforcement practices in the future.

## Figures and Tables

**Table 1.** Range of the factors utilized to evaluate the distraction index.

Factors	Range of Factors

Attention time (*AT*)	The time during which the driver focuses on the road.
Response time (*RT*)	Up to 85% of drivers respond within 0.75 s, and 95% of drivers respond within 1.5 s [34].
Glance duration (*GD*)	The duration for which the driver shifts their gaze to the equipment when requested to perform a specific task.
Hand movement (*HM*)	The duration at which the driver grips or releases their hands on the steering wheel.
Interaction frequency with onboard devices (*IF*)	The frequency for which the driver shifts their gaze to the onboard devices.

**Table 2.** The four designated routine tasks performed by participants.

Task	Range of Tasks
Task #1	The participant drives with the left hand while reaching out with the right hand from the steering wheel to touch two spots on the monitor of a passenger seat-mounted or center console-mounted MDT. When the task is complete, the participant returns the right hand to the original position on the steering wheel.
Task #2	The participant drives with the left hand while reaching out with the right hand from the steering wheel to the keyboard of the passenger seat-mounted or center console-mounted MDT and enters a six-digit input (three letters followed by three numbers). When the task is complete, the participant returns the right hand to the original position on the steering wheel.
Task #3	The participant drives with the left hand while reaching out with the right hand from the steering wheel to the radio unit to perform a zone selection task. This task includes pressing the zone button, pressing (three times) the navigation button to scroll the zone to the right, and pressing the home button to confirm the desired zone. When the task is complete, the participant returns the right hand to the original position on the steering wheel.
Task #4	The participant drives with the left hand while reaching out with the right hand from the steering wheel to the light and siren control unit to perform a light and siren operation task. The task includes flipping the color selection switch from red to blue and then pressing the MAN button three times. When the task is complete, the participant returns the right hand to the original position on the steering wheel.

**Table 3.** Sequence of the assigned tasks and the recorded events.

Task	Event Description	Time Stamp

Task 1	Task 1 buzzer start	
	Data collection start—Remove the hand from the steering wheel	
	Red spot 1 triggered	
	Red spot 2 triggered	
	Data collection end—Return the hand back to the steering wheel	

Task 2	Task 2 buzzer start	
	Data collection start—Remove the hand from the steering wheel	
	Data collection end—Return the hand back to the steering wheel	

Task 3	Task 3 buzzer start	
	Zone switch triggered	
	Navigation switch triggered	
	Home switch triggered	
	Data collection end—Return the hand back to the steering wheel	

Task 4	Task 4 buzzer start	
	Data collection start—Remove the hand from the steering wheel	
	Sliding switch 01 triggered	
	Sliding switch 02 triggered	
	Red alley light push button triggered	
	Blue alley light push button triggered	
	Data collection end—Return the hand back to the steering wheel	

**Table 4.** Five samples from the 200 sets of emulated data of driver responses.

#	Attention Time Percent (%)	Glance Duration (s)	Hand Movement Time (s)	Interaction Frequency (Times)	Response Time (s)	Distraction Index

1	81.16	0.18	0	0	0.87	False
2	56.46	4.29	4.02	7	1.45	True
3	85.24	0.18	0	0	1.23	False
4	67.16	4.68	5.56	6	0.77	True
5	68.95	2.96	7.71	11	1.01	True

## Data Availability

The data presented in this study are available on request from the corresponding author.

## Abbreviations

The following abbreviations are used in this manuscript:

DVI: Driver–Vehicle Interface
LEO: Law Enforcement Officers
UDP: User Datagram Protocol
MDT: Mobile Data Terminal
AOI: Area of Interest
AT: Attention Time
RT: Response Time
GD: Glance Duration
HM: Hand Movement
IF: Interaction Frequency with Onboard Devices
DI: Distraction Index
